# Testing for differences in distribution tails to test for differences in 'maximum' lifespan

**DOI:** 10.1186/1471-2288-8-49

**Published:** 2008-07-25

**Authors:** Guimin Gao, Wen Wan, Sijian Zhang, David T Redden, David B Allison

**Affiliations:** 1 Department of Biostatistics, Section on Statistical Genetics, University of Alabama at Birmingham, Birmingham, Alabama, USA; 2 Department of Nutrition Sciences, University of Alabama at Birmingham, Birmingham, Alabama, USA; 3 Clinical Nutrition Research Center, University of Alabama at Birmingham, Birmingham, Alabama, USA; 4 Biostatistics and Bioinformatics Unit, Comprehensive Cancer Center, University of Alabama at Birmingham, Birmingham, Alabama, USA; 5 Department of Biostatistics, University of Alabama at Birmingham, Birmingham, Alabama, USA

## Abstract

**Background:**

Investigators are actively testing interventions intended to increase lifespan and wish to test whether the interventions increase maximum lifespan. Based on the fact that one cannot be assured of observing population maximum lifespans in finite samples, in previous work, we constructed and validated several tests of difference in the upper parts of lifespan distributions between a treatment group and a control group by testing whether the probabilities that observations are above some threshold defining 'old' or being in the tail of the survival distribution are equal in the two groups. However, a limitation of these tests is that they do not consider *how much *above the threshold any particular observation is.

**Methods:**

In this article we propose new methods which improve upon our previous tests by considering not only whether an observation is above some threshold, but also the magnitudes by which observations exceed the threshold.

**Results:**

Simulations show that the new methods control type I error rates quite well and that the power of the new methods is usually higher than that of the tests we previously proposed. In illustrative analyses of two real datasets involving rodents, when setting the threshold equal to 110 (100) weeks for the first (second) datasets, the new methods detected differences in 'maximum lifespan' between groups at nominal alpha levels of 0.01 (0.05) for the first (second) datasets and provided more significant results than competitor tests.

**Conclusion:**

The new methods not only have good performance in controlling the type I error rates but also improve the power compared with the tests we previously proposed.

## Background

Investigators are actively testing interventions intended to increase lifespan [1]. Caloric restriction (CR) is the intervention most well established as able to increase lifespan in experimental models [2], and investigators are now seeking other interventions that may mimic the life-prolonging effects of CR without requiring a reduction in caloric intake [3]. It is frequently said that CR not only increases average lifespan, but also 'maximum' lifespan [4]. Many researchers in the field of aging therefore wish to test whether other interventions increase maximum lifespan.

Recognizing this and the fact that one cannot be assured of observing population maximum lifespans in finite samples, Wang et al. [5] constructed and validated several tests (hereafter, the *'Wang-Allison tests'*) of differences in the upper parts of lifespan distributions by building on the work of Redden et al. [6] in the area of quantile regression. Wang et al. also showed that a commonly used test for differences in maximum lifespan that involved comparing the means of the top *p*% (e.g., top 10%) of each of two samples (e.g., a treatment and a control sample) was not valid in that it had an excessive type-1 error rate. Nevertheless, there is appeal to using the full continuity of information in the upper tails of the sample distribution, and colleagues have recently suggested to us that a limitation of the Wang-Allison tests is that they only treat individual lifespans as being above or below some threshold defining 'old' or being in the tail of the survival distribution. That is, the Wang-Allison tests do not consider *how much *above the threshold any particular observation is, only *whether *the observation is above the threshold. We acknowledge this limitation and in response, we herein develop new tests that utilize the continuity of information among observations that exceed the threshold of interest, are more powerful than competing tests, including the Wang-Allison tests, in most cases, and remain valid under the null hypothesis of no effect on 'maximum' lifespan.

## Methods

### Development of the tests

Consider an experiment with two groups, *treatment *and *control*. The extension to more than two groups is straightforward (see discussion section). Let *X *be an indicator variable taking the value 1 for observations in the treatment group and 0 for observations in the control group. Let *Y *denote survival time. Let *τ *denote some threshold chosen by the investigator to denote an extreme portion of the distribution. In survival studies, *τ *can be chosen in advance to correspond to an age considered 'old' (e.g., 30 months in mice) or set to some high sample percentile (e.g., the 90th). Critically important, *τ *must be set to the same value for the two groups. That is, if *τ *is to be defined by an upper sample quantile, it should be the upper sample quantile of both of the two groups combined, not of each group separately.

Although not described in exactly these terms in the paper by Wang et al. [5], the Wang-Allison tests essentially create a new variable, *W*, where for the i^th ^subject, *W*_*i *_≡ 0 if *Y*_*i *_≤ *τ*, and *W*_*i *_≡ 1 if *Y*_*i *_> *τ*, and subsequently tests whether *W *is associated with *X *using an appropriate test statistic.

Thus, the Wang-Allison tests test the following null hypothesis:

*H*_0,*A *_: *P *(*Y *> *τ*|*X *= 1) = *P*(*Y *> *τ*|*X *= 0).

**Figure 1:**
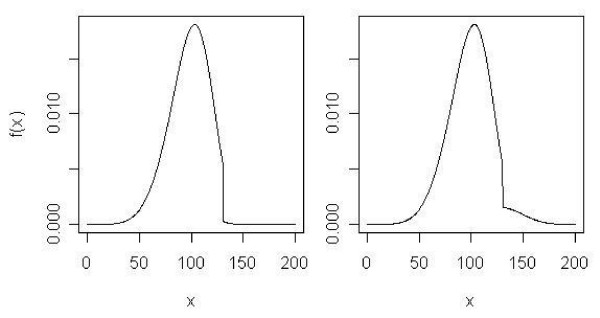
The left graph is the density for control group (X = 0), 0.9*Weibull(5.73, 106.6)*I(X ≤ 130) + 0.1*Weibull(5.40, 100.06)*I(X > 130), and the right graph is the density for treatment group (X = 1), 0.9*Weibull(5.73, 106.6)*I(X ≤ 130) + 0.1*Weibull(5.45, 130.06)*I(X > 130), where *P*(*Y *> *τ*|*X *= 1) = *P*(*Y *> *τ*|*X *= 0) and yet the average magnitude by which lifespans exceed *τ *when X = 1 is different than when X = 0. *τ *is 90^th ^percentile of the all observations in treatment and control groups.

A problem with the Wang-Allison tests is that, hypothetically, *P *(*Y *> *τ*|*X *= 1) may equal *P *(*Y *> *τ*|*X *= 0) and yet the average magnitude by which lifespans exceed *τ *when X = 1 may be radically different than when X = 0. This is exemplified in the hypothetical frequency distributions depicted in Figure 1. Note that these hypothetical distributions are not intended to be realistic, but only to clarify the point.

Let *X*^1 ^and *X*^0 ^denote the numbers of observations with *Y*_*i *_> *τ *in the treatment group and control group, respectively. The Wang-Allison tests use the test procedures for two independent binomial proportions [7] and these procedures require that *X*^1 ^and *X*^0 ^are independent. In the Wang-Allison tests, if the threshold is set in advance according to prior knowledge, *X*^1 ^and *X*^0 ^can satisfy the requirement of independence. But if *τ *is set to be the 90-th percentile, *X*^1 ^and *X*^0 ^may not be independent, this creates a theoretical problem. However, on an empirical level, our simulations show that in the sample sizes we considered, this is not an apparent problem because the Wang-Allison tests have very high power and can control type I error quit well in the simulation studies and are practical for the lifespan studies). When *X*^1 ^and *X*^0 ^are not independent, simulation studies (including estimation of power and type I error) are an effective way to evaluate the methods (such as Wang-Allison tests) using the test procedures for two independent binomial proportions.

**Figure 2:**
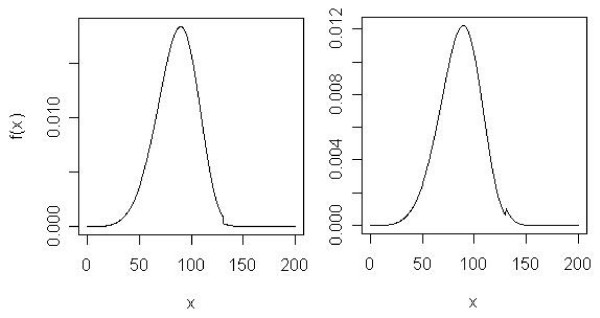
The left graph is the density for control group (X = 0), 0.9*Weibull(5.07, 93.52)*I(X ≤ 130) + 0.1*Weibull(5.40, 100.06)*I(X > 130), and the right graph for treatment group (X = 1), 0.6*Weibull(5.07, 93.52)*I(X ≤ 130) + 0.4*Weibull(5.40, 100.06)*I(X > 130), where *P*(*Y *> *τ*|*X *= 1) ≠ *P*(*Y *> *τ*|*X *= 0), *μ *(*Y *|*Y *> *τ *∩ *X *= 1) = *μ *(*Y *|*Y *> *τ *∩ *X *= 0), and *μ *(•) denotes the population mean of (•). *τ *is 90^th ^percentile of the all observations in treatment and control groups.

An alternative to testing *H*_0,*A *_is to test the following conceptually related but mathematically distinct null hypothesis:

*H*_0,*B *_: *μ *(*Y*|*Y *> *τ *∩ *X *= 1) = *μ *(*Y*|*Y *> *τ *∩ *X *= 0),

where *μ *(•) denotes the population mean (or expectation) of (•). Though appealing, a problem with testing *H*_0,*B *_is that when *P *(*Y *> *τ*|*X *= 1) >> *P *(*Y *> *τ*|*X *= 0) or *P *(*Y *> *τ*|*X *= 1) <<*P *(*Y *> *τ*|*X *= 0), for any finite sample with equal initial assignment to the two groups, *E *[*n*_0_] <<*E *[*n*_1_] or *E *[*n*_0_] >> *E *[*n*_1_], where *E *[*n*_0_] denotes the expected number of observations in the control group for which *Y *> *τ*, and *E *[*n*_1_] denotes the expected number of observations in the treatment group for which *Y *> *τ*. This imbalance between *E *[*n*_0_] and *E *[*n*_1_] will greatly reduce the power to reject *H*_0,*B*_. In fact, in the extreme, when either *P *(*Y *> *τ*|*X *= 1) or *P *(*Y *> *τ*|*X *= 0), there will be zero power to reject *H*_0,*B *_(actually, it is appropriate to say that *H*_0,*B *_is undefined in such cases). Such a situation is exemplified in the hypothetical frequency distributions depicted in Figure 2. Again, these hypothetical distributions are not intended to be realistic, but only to clarify the point.

Thus, one can conceive situations in which the power to reject *H*_0,*A *_will be zero and yet the upper tails of the distribution are clearly different. Similarly, one can conceive situations in which the power to reject *H*_0,*B *_will be zero and yet again the upper tails of the distribution are clearly different. Hence, we propose a single-step union-intersection test [8] of the following compound null hypothesis:

*H*_0,*C *_: [*P*(*Y *> *τ*|*X *= 1) = *P*(*Y *> *τ*|*X *= 0)] ∩ [*μ*(*Y*|*Y *> *τ *∩ *X *= 1) = *μ *(*Y*|*Y *> *τ *∩ *X *= 0)].

We construct the test of *H*_0,*C *_with the following simple procedure. Define a new variable *Z *such that *Z*_*i *_≡ *I*(*Y*_*i *_> *τ*)*Y*_*i*_, where *I*(•) denotes the indicator function taking on values of one if (•) is true and zero otherwise. One can then simply conduct an appropriate test (several candidates will be considered below) of whether the population mean of Z is significantly different between the treatment and control groups. This approach (hereafter new *tests*), has several desirable properties. First and foremost, when an appropriate test statistic is used, the approach will be valid. That is, unlike the conditional t-tests (CTTs) commonly used and shown to be invalid by Wang et al. [5], when *H*_0,*C *_is true, it will only be rejected 100*α% of the time at the nominal *α *level even if *f*(*Y*|*Y *≤ *τ *∩ *X *= 1) ≠ *f*(*Y*|*Y *≤ *τ *∩ *X *= 0), where *f*(•) denotes the probability density function of (•).

Note that expectation (or population mean) of *Z*, *μ*(*Z*) = *P*(*Y *> *τ*) *μ*(*Y *| *Y *> *τ*). Therefore the new test for *H*_0,*C *_is really testing whether

*P*(*Y *> *τ *| *X *= 1) *μ *(*Y *| *Y *> *τ *∩ *X *= 1) = *P*(*Y *> *τ *| *X *= 0) *μ *(*Y *| *Y *> *τ *∩ *X *= 0),

while the method for *H*_0,*B *_is testing whether *μ *(*Y *| *Y *> *τ *∩ *X *= 1) = *μ*(*Y *| *Y *> *τ *∩ *X *= 0) and the method for *H*_0,*A *_is testing whether *P*(*Y *> *τ *| *X *= 1) = *P*(*Y *> *τ *| *X *= 0). The mean difference of *μ*(*Z*) between two groups consists of two components: the difference between probabilities *P*(*Y *> *τ *| *X *= 1) and *P*(*Y *> *τ *| *X *= 0) and the difference between expectations *μ *(*Y *| *Y *> *τ *∩ *X *= 1) and *μ *(*Y *| *Y *> *τ *∩ *X *= 0). The test for *H*_0,*A *_focuses on the first component and the test for *H*_0,*A *_focuses on the second one, while the test for *H*_0,*C *_is related to both components.

We also note that Dominici and Zeger [9] studied similar mean difference components for two groups (cases and controls) by estimating the mean difference Δ(***v***) for the two groups conditional on a vector of covariates ***v ***for zero-inflated data through smooth quantile ratio estimation with regression,

Δ(***v***) = *P*(*Y *> 0 | *X *= 1, ***v***) *μ *(*Y *| *Y *> 0, *X *= 1, ***v***) - *P*(*Y *> 0| *X *= 0, ***v***) *μ *(*Y *| *Y *> 0, *X *= 0, ***v***),

where, *Y *is nonnegative random variable denoting the health expenditures. While Dominici and Zeger [9] estimate the mean difference of nonnegative random variables (*Y*) for two groups, our methods test the mean difference of random variables (*Y*) which are greater than threshold *τ*.

### Evaluation of the tests

We evaluate the tests via computer simulation. For each scenario simulated, we evaluate the tests at the 2-tailed .05 *α *level and at the 2-tailed .01 *α *level using 5,000 simulated datasets per scenario (except for permutation tests where we use 1,000 datasets per scenario and 1,000 random permutations by Monte Carlo sampling for each dataset). In simulation 1, we first evaluate performance in simulation under the null hypothesis *H*_0,*C *_(i.e., both *H*_0,*A *_and *H*_0,*B *_are true) and yet *f *(*Y*|*Y *≤ *τ *∩ *X *= 1) is radically different from *f *(*Y*|*Y *≤ *τ *∩ *X *= 0). After showing that the tests remain valid even in these extreme circumstances, we compare their power in several scenarios (simulations 2–4) described below. For each scenario, we assumed that there were two groups with an equal number of subjects per group. We ran scenarios with 50, 80, or 100 subjects in each of the two groups, realistic sample sizes for animal model longevity research.

**Figure 3:**
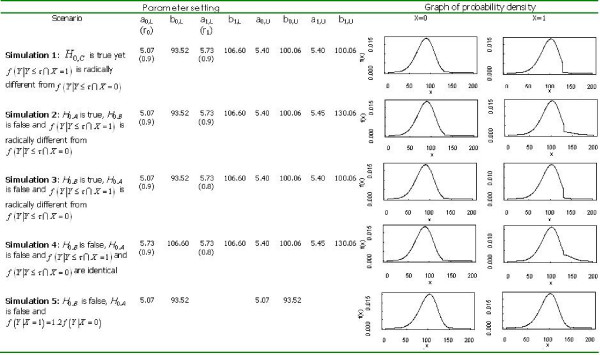
Parameter values and distributions for component Weibull distributions used in each simulation.

We simulated data using a concatenation of Weibull distributions to flexibly emulate the data observed in a real study [10] of obese animals (control; X = 0) versus animals that were obese and then lost weight via CR (treatment; X = 1). Specifically, For example, in simulations 1–4, we simulated Y from the following distribution:

$$f(Y|X=j)=r_{j}\left[\frac{b_{j,L}}{a_{j,L}}{\left(\frac{Y}{a_{j,L}}\right)}^{b_{j,L}-1}e^{-{\left(\frac{Y}{a_{j,L}}\right)}^{b_{j,L}}}\right]I(Y\leq 130)+(1-r_{j})\left[\frac{b_{j,U}}{a_{j,U}}{\left(\frac{Y}{a_{j,U}}\right)}^{b_{j,U}-1}e^{-{\left(\frac{Y}{a_{j,U}}\right)}^{b_{j,U}}}\right]I(Y>130),$$

where j = 0 to 1, lifespan (Y) is measured in weeks, a_j,L _and b_i,L _are the parameters of a Weibull distribution for the lower 90% of the distribution, and a_j,U _and b_i,U _are the parameters of a Weibull distribution for the upper 10% of the distribution. *r*_*j *_is a proportion parameter, for example *r*_*j *_= 0.9. The specific values of the parameters used are provided in Figure 3.

### Delineation of tests to be evaluated

Each of the tests listed below was implemented in two manners, first with *τ *set in advance to a fixed lifespan value (130 weeks), and second with *τ *set at the sample 90^th ^percentile of the two groups combined. In real-life situations, one usually *does *know the threshold of interest *a priori*. We do recognize that we will not have such knowledge in all cases. It is for this reason that when analyzing the simulated data, we also consider a threshold of the 90^th ^percentile of the data allowing for an *ad hoc *data-based determination of a threshold.

#### Tests of H_0,A _(Wang-Allison tests)

For comparative purposes, the first category of tests we evaluated were the tests denoted QT3 and QT4 in Wang et al [5] which are, respectively, Boschloo's test and an exact unconditional test based on the observed difference divided by its estimated standard error under the null hypothesis (score statistic) and are described in more detail by Mehrotra et al. [7]. These were the two tests that Wang et al. [5] had found performed best as tests of *H*_0,*A*_.

#### Tests of H_0,B_

In testing *H*_0,*B*_, subjects were only included in the analysis when their lifespans exceeded *τ*. Distributions of survival times (lifespans) are rarely Gaussian and, even if they were nearly Gaussian after, for example, log transformation, the distribution of just the tail portion (i.e., *f *(*Y*|*Y *> *τ*) would not be. Hence, in constructing tests we relied on nonparametric statistical methods. Specifically, we used the Wilcoxon-Mann-Whitney (exact) test [11,12] and a permutation test (with t-statistic) as described by Good [13] to test for differences in lifespan among those subjects whose lifespans exceeded *τ*.

#### Tests of H_0,C _(new tests)

In testing *H*_0,*C*_, all subjects were analyzed, but the variable analyzed was Z as defined above. Because the distribution of Z cannot be normally distributed, we again used the Wilcoxon-Mann-Whitney test and a permutation test to test for differences in Z.

For a dataset with *n*_1 _(*n*_2_) subjects in treatment (control) group, the permutation test can be performed in the following way: First put all the (*n*_1 _+*n*_2_) subjects together, and then generate 1000 replicated datasets. For each replicated dataset, we randomly sample *n*_1 _subjects from the (*n*_1 _+*n*_2_) subjects and assign them to treatment group, and assign the left *n*_2 _subjects to control group. We run T-test on the observed dataset and the 1000 replicated datasets. Let *T*_0 _be the T value for the observed dataset, then p-value for the permutation test is calculated as the proportion of replicated datasets with absolute T values greater than or equal to the absolute valued of *T*_0_.

## Results

Results are displayed in Tables 1 to 5. As can be seen, the new methods for tests of *H*_0,*C *_controls type I error rates quite well. The power of the new methods are always higher than or very close to that of the methods for tests of *H*_0,*A *_(Wang-Allison tests) and are higher than that of the methods for tests of *H*_0,*B *_(Wilcoxon-Mann-Whitney tests and permutation tests for observations above the threshold *τ*) in some of the simulations.

**Table 1:** Performance (type 1 error rates) of the tests in simulation 1 under *H*_0,*C *_(i.e., both *H*_0,*A *_and *H*_0,*B *_are true) and yet *f *(*Y*|*Y *≤ *τ *∩ *X *= 1) is radically different from *f *(*Y*|*Y *≤ *τ *∩ *X *= 0) (see Figure 3 for details of simulation).

**Test**	**Sample Size (N) Per Group**					
	
	50		80		100	
			
	*α *= .05	*α *= .01	*α *= .05	*α *= .01	*α *= .05	*α *= .01
***Tests of H *_**0,*A ***_*(Wang-Allison tests)***						
QT3 with *τ *set to 130.	0.032 (.027, .036)^#^	0.008 (.005, .011)	0.041 (.036, .046)	0.006 (.003, .009)	0.040 (.035, .045)	0.006 (.003, .009)
QT3 with *τ *set to sample 90^th ^percentile	0.026 (.022, .030)	0.026* (.020, .032)	**0.080 **(.072, .088)	0.007 (.004, .010)	0.040 (.035, .045)	0.010 (.006, .014)
QT4 with *τ *set to 130.	0.038 (.033, .043)	0.008 (.005, .011)	0.051 (.045, .057)	0.009 (.006, .012)	0.047 (.041, .053)	0.007 (.004, .010)
QT4 with *τ *set to sample 90^th ^percentile.	0.026 (.022, .030)	**0.026 **(.020, .032)	**0.083 **(.075, .091)	**0.026 **(.020, .032)	0.040 (.035, .045)	0.010 (.006, .014)
***Tests of H*_**0,*B***_**						
Wilcoxon-Mann-Whitney** with *τ *set to 130.	0.041 (.036, .046)	**0.017 **(.012, .022)	0.044 (.038, .050)	0.008 (.005, .011)	0.046 (.040, .052)	0.008 (.005, .011)
Wilcoxon-Mann-Whitney with *τ *set to sample 90^th ^percentile.	0.049 (.043, .055)	0.014 (.010, .018)	**0.065 **(.058, .072)	**0.015 **(.011, .019)	**0.080 **(.072, .088)	**0.018 **(.013, .023)
Permutation test with *τ *set to 130.	0.050 (.036, .064)	0.009 (.001, .017)	0.050 (.036, .064)	0.011 (.002, .020)	0.064 (.049, .079)	0.015 (.005, .025)
Permutation test with *τ *set to sample 90^th ^percentile.	**0.077 **(.060, .094)	0.016 (.006, .026)	**0.078 **(.061, .095)	0.022 (.010, .034)	**0.083 **(.066, .100)	0.019 (.008, .030)
***Tests of H*_**0,*C ***_*(new tests)***						
Wilcoxon-Mann-Whitney with *τ *set to 130.	0.042 (.036, .048)	0.007 (.004, .010)	0.049 (.043, .055)	0.010 (.006, .014)	0.051 (.045, .057)	0.008 (.005, .011)
Wilcoxon-Mann-Whitney with *τ *set to sample 90^th ^percentile.	0.055 (.049, .061)	**0.015 **(.011, .019)	**0.060 **(.053, .067)	**0.015 **(.011, .019)	**0.061 **(.054, .068)	**0.015 **(.011, .019)
Permutation test with *τ *set to 130.	0.052 (.038, .066)	0.015 (.005, .025)	0.047 (.034, .060)	0.009 (.001, .017)	0.057 (.043, .071)	0.007 (.000, .014)
Permutation test with *τ *set to sample 90^th ^percentile.	0.045 (.032, .058)	0.017 (.006, .028)	0.062 (.047, .077)	0.011 (.002, .020)	**0.068 **(.053, .084)	0.018 (.007, .029)

^#^2-tailed 95% confidence interval.

*The bolded values are those simulated type I error rates which are significantly higher than the nominal *α *at the 2-tailed 95% confidence level (i.e., the lower bound of the interval is higher than *α*). Note that for the permutation tests we used 1000 replicated datasets and for other tests we used 5000 replicated datasets.

**In all the simulation studies (Tables 1-5), we used Wilcoxon-Mann-Whitney exact test.

**Table 2:** Performance of the tests in simulation 2, *H*_0,*A *_is true, *H*_0,*B *_is false and *f *(*Y*|*Y *≤ *τ *∩ *X *= 1) is radically different from *f *(*Y*|*Y *≤ *τ *∩ *X *= 0) (see Figure 3 for details of simulation).

**Test**	**Sample Size (N) Per Group**					
	
	50		80		100	
			
	*α *= .05	*α *= .01	*α *= .05	*α *= .01	*α *= .05	*α *= .01
***Tests of H*_**0,*A ***_*(Wang-Allison tests)***						
QT3 with *τ *set to 130.	0.032	0.008	0.041	0.006	0.040	0.006
QT3 with *τ *set to sample 90^th ^percentile.	0.034	0.034	0.104	0.009	0.062	0.018
QT4 with *τ *set to 130.	0.038	0.008	0.051	0.009	0.047	0.007
QT4 with *τ *set to sample 90^th ^percentile.	0.034	0.034	0.104	0.033	0.062	0.018
***Tests of H*_**0,*B***_**						
Wilcoxon-Mann-Whitney with *τ *set to 130.	0.264	0.090	0.504	0.261	0.631	0.368
Wilcoxon-Mann-Whitney with *τ *set to sample 90^th ^percentile.	0.16	0.051	0.314	0.143	0.406	0.220
Permutation test with *τ *set to 130	0.337	0.111	0.608	0.332	0.737	0.456
Permutation test with *τ *set to sample 90^th ^percentile.	0.197	0.047	0.423	0.204	0.525	0.284
***Tests of H*_**0,*C ***_*(new tests)***						
Wilcoxon-Mann-Whitney with *τ *set to 130.	0.051	0.008	0.062	0.012	0.056	0.010
Wilcoxon-Mann-Whitney with *τ *set to sample 90^th ^percentile.	0.107	0.029	0.090	0.028	0.124	0.035
Permutation test with *τ *set to 130.	0.061	0.013	0.055	0.012	0.065	0.014
Permutation test with *τ *set to sample 90^th ^percentile.	0.109	0.032	0.097	0.03	0.129	0.046

**Table 3:** Performance of the tests in simulation 3, *H*_0,*B *_is true, *H*_0,*A *_is false and *f *(*Y*|*Y *≤ *τ *∩ *X *= 1) is radically different from *f *(*Y*|*Y *≤ *τ *∩ *X *= 0) (see Figure 3 for details of simulation).

**Test**	**Sample Size (N) Per Group**					
	
	50		80		100	
			
	*α *= .05	*α *= .01	*α *= .05	*α *= .01	*α *= .05	*α *= .01
***Tests of H*_**0,*A ***_*(Wang-Allison tests)***						
QT3 with *τ *set to 130.	0.244	0.101	0.412	0.181	0.490	0.258
QT3 with *τ *set to sample 90^th ^percentile.	0.102	0.102	0.332	0.051	0.297	0.143
QT4 with *τ *set to 130.	0.266	0.102	0.418	0.187	0.514	0.274
QT4 with *τ *set to sample 90^th ^percentile.	0.102	0.102	0.332	0.151	0.297	0.143
***Tests of H*_**0,*B***_**						
Wilcoxon-Mann-Whitney with *τ *set to 130.	0.046	0.013	0.049	0.011	0.045	0.008
Wilcoxon-Mann-Whitney with *τ *set to sample 90^th ^percentile.	0.048	0.019	0.044	0.01	0.041	0.009
Permutation test with *τ *set to 130.	0.042	0.007	0.046	0.012	0.064	0.013
Permutation test with *τ *set to sample 90^th ^percentile.	0.046	0.009	0.046	0.012	0.044	0.01
***Tests of H*_**0,*C ***_*(new tests)***						
Wilcoxon-Mann-Whitney with *τ *set to 130.	0.276	0.111	0.420	0.201	0.517	0.271
Wilcoxon-Mann-Whitney with *τ *set to sample 90^th ^percentile.	0.182	0.07	0.278	0.104	0.35	0.154
Permutation test with *τ *set to 130.	0.291	0.101	0.427	0.203	0.515	0.28
Permutation test with *τ *set to sample 90^th ^percentile.	0.169	0.067	0.264	0.107	0.363	0.173

**Table 4:** Performance of the tests in simulation 4, *H*_0,*B *_is false, *H*_0,*A *_is false and *f *(*Y*|*Y *≤ *τ *∩ *X *= 1) and *f *(*Y*|*Y *≤ *τ *∩ *X *= 0) are identical (see Figure 3 for details of simulation).

**Test**	**Sample Size (N) Per Group**					
	
	50		80		100	
			
	*α *= .05	*α *= .01	*α *= .05	*α *= .01	*α *= .05	*α *= .01
***Tests of H*_**0,*A ***_*(Wang-Allison tests)***						
QT3 with *τ *set to 130.	0.244	0.101	0.412	0.181	0.490	0.258
QT3 with *τ *set to sample 90^th ^percentile.	0.363	0.363	0.735	0.337	0.753	0.600
QT4 with *τ *set to 130.	0.266	0.102	0.418	0.187	0.514	0.274
QT4 with *τ *set to sample 90^th ^percentile.	0.363	0.363	0.735	0.555	0.753	0.600
***Tests of H_**0,*B***_***						
Wilcoxon-Mann-Whitney with *τ *set to 130.	0.409	0.172	0.684	0.411	0.804	0.56
Wilcoxon-Mann-Whitney with *τ *set to sample 90^th ^percentile.	0.245	0.142	0.33	0.144	0.434	0.176
Permutation test with *τ *set to 130.	0.517	0.244	0.81	0.568	0.913	0.728
Permutation test with *τ *set to sample 90^th ^percentile.	0.169	0.039	0.428	0.190	0.569	0.249
***Tests of H*_**0,*C ***_*(new tests)***						
Wilcoxon-Mann-Whitney with *τ *set to 130.	0.374	0.171	0.528	0.280	0.629	0.373
Wilcoxon-Mann-Whitney with *τ *set to sample 90^th ^percentile.	0.602	0.353	0.734	0.552	0.865	0.724
Permutation test with *τ *set to 130.	0.393	0.177	0.524	0.288	0.626	0.377
Permutation test with *τ *set to sample 90^th ^percentile.	0.619	0.365	0.726	0.553	0.852	0.704

**Table 5:** Performance of the tests in simulation 5, *H*_0,*B *_is false, *H*_0,*A *_is false and *f *(*Y*|*X *= 1) = 1.2*f *(*Y*|*X *= 0) (see Figure 3 for details of simulation).

**Test**	**Sample Size (N) Per Group**					
	
	50		80		100	
			
	*α *= .05	*α *= .01	*α *= .05	*α *= .01	*α *= .05	*α *= .01
***Tests of H*_**0,*A ***_*(Wang-Allison tests)***						
QT3 with *τ *set to 130.	0.663	0.349	0.925	0.754	0.965	0.883
QT3 with *τ *set to sample 90^th ^percentile.	0.815	0.815	0.996	0.885	0.997	0.986
QT4 with *τ *set to 130.	0.765	0.349	0.941	0.797	0.981	0.906
QT4 with *τ *set to sample 90^th ^percentile.	0.815	0.815	0.996	0.969	0.997	0.986
***Tests of H*_**0,*B***_**						
Wilcoxon-Mann-Whitney with *τ *set to 130.	0.001	0.000	0.006	0.000	0.010	0.000
Wilcoxon-Mann-Whitney with *τ *set to sample 90^th ^percentile.	0.016	0.000	0.035	0.002	0.058	0.009
Permutation test with *τ *set to 130	0.001	0.000	0.036	0.003	0.061	0.005
Permutation test with *τ *set to sample 90^th ^percentile.	0.032	0.002	0.082	0.017	0.124	0.041
***Tests of H*_**0,*C ***_*(new tests)***						
Wilcoxon-Mann-Whitney with *τ *set to 130.	0.556	0.239	0.920	0.742	0.979	0.897
Wilcoxon-Mann-Whitney with *τ *set to sample 90^th ^percentile.	0.932	0.767	0.995	0.964	0.999	0.992
Permutation test with *τ *set to 130.	0.852	0.646	0.960	0.850	0.993	0.940
Permutation test with *τ *set to sample 90^th ^percentile.	0.942	0.786	0.995	0.958	0.997	0.986

Table 1 shows the type I error rate of the tests (in simulation 1) when the null hypothesis *H*_0,*C *_is true (i.e., both *H*_0,*A *_and *H*_0,*B *_are true) and yet *f *(*Y*|*Y *≤ *τ *∩ *X *= 1) is radically differentfrom *f *(*Y*|*Y *≤ *τ *∩ *X *= 0). The type I error rates of the new methods are comparable to those of the methods for tests of *H*_0,*A *_and those of the methods for tests of *H*_0,*B *_. It is note worthy that there is a slight but fairly consistent excess of type I errors when the sample 90^th ^percentile is used rather than a fixed cutoff point. This is because the sample 90^th ^percentile is a random variable and when it falls below its population level, the null hypothesis is no longer strictly true in our simulations. That is, the tests remain valid tests of differences in distributions above the actual value used but should not be strictly interpreted as tests of differences in distributions above the 90^th ^(or any other percentile). In practice, this distinction is probably trivial.

In simulation 2 (see Table 2), where *H*_0,*A *_is true, *H*_0,*B *_is false and *f *(*Y*|*Y *≤ *τ *∩ *X *= 1) is radically different from *f *(*Y*|*Y *≤ *τ *∩ *X *= 0), the new methods for tests of *H*_0,*C *_and the methods for tests of *H*_0,*A *_have lower power than that of the corresponding methods for tests of *H*_0,*B*_, however, the new methods for tests of *H*_0,*C *_can slightly improve the power compared to the methods for tests of *H*_0,*A*_.

Table 3 shows the power of the tests in Simulation 3, where *H*_0,*B *_is true, *H*_0,*A *_is false and *f *(*Y*|*Y *≤ *τ *∩ *X *= 1) is radically different from *f *(*Y*|*Y *≤ *τ *∩ *X *= 0). The new methods for tests of *H*_0,*C *_and the methods for tests of *H*_0,*A *_have very similar power which is much higher than that of the corresponding methods for tests of *H*_0,*B*_.

From simulation 4 (see Table 4), where *H*_0,*B *_is false, *H*_0,*A *_is false and *f *(*Y*|*Y *≤ *τ *∩ *X *= 1) and *f *(*Y*|*Y *≤ *τ *∩ *X *= 0) are identical, we can find that the new methods for tests of *H*_0,*C *_always have higher power than the corresponding methods for tests of *H*_0,*A*_. When *τ *being set to the 90th percentile of the sample, the new methods also have higher power than the corresponding methods for tests of *H*_0,*B*_.

Finally, we conducted a set of simulations under what we perceived to be the most realistic situations. Here both *H*_0,*A *_and *H*_0,*B *_are false, *f *(*Y*|*Y *≤ *τ *∩ *X *= 1) is quite different from *f *(*Y*|*Y *≤ *τ *∩ *X *= 0), and the distributions have no discontinuities. In other words, there is just a simple reduction in the hazard rate when X = 1. Table 5 presents the power of the tests in Simulation 5, where *f *(*Y*|*X *= 1) = 1.2*f *(*Y*|*X *= 0). In this simulation, the methods for tests of *H*_0,*B *_almost have no power because the control group always has no or few observations above the threshold *τ *. The new methods for tests of *H*_0,*C*_, when using a permutation test, have power higher than or equal to that of the methods for tests of *H*_0,*A*_.

### Illustration with real data

**Figure 4:**
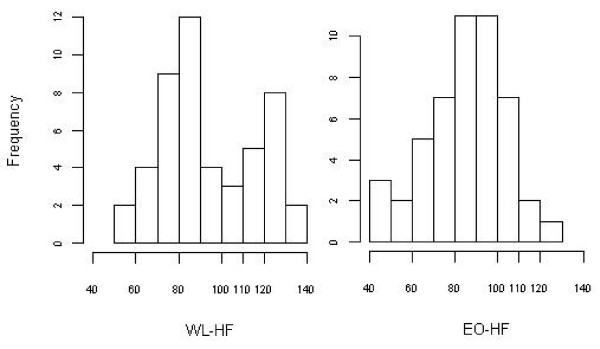
The left (right) graph is the histogram of lifespan for WL-HF (EO-HF) group in the data from Vasselli et al. [10].

**Figure 5:**
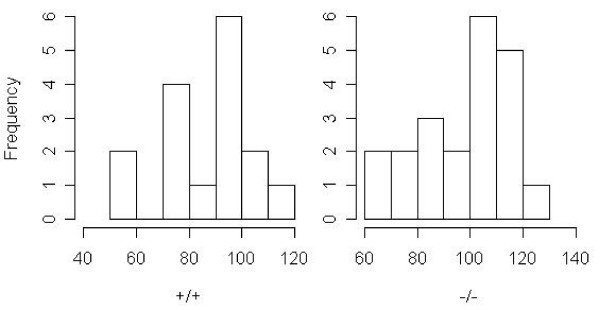
The left (right) graph is the histogram of lifespan for group with genotype '+/+' ('-/-') in the data from Redmann & Argyropoulos [14].

To illustrate the methods, we applied them to two real datasets. In both of these datasets, prior research had shown differences in overall survival rate and we tested for differences in 'maximum lifespan' herein. The first was a subset of data reported by Vasselli et al [10]. The subset of the data consists of two groups of Sprague-Dawley rats, those kept on a high-fat diet ad libitum throughout life and becoming obese (EO-HF) and those kept on a high-fat diet ad libitum until early-middle adulthood, becoming obese, and subsequently reduced to normal weight via caloric restriction, but on the same high-fat diet (WL-HF). Each group had 49 rats (see Figure 4 for the histograms for the data). The second dataset was from a study comparing the lifespan of Agouti-related protein-deficient (AgRP(-/-)) mice to wildtype mice (+/+) as reported by Redmann & Argyropoulos [14]. This dataset consists of 16 mice with genotype '+/+' and 21 mice with genotype '-/-' (see Figure 5 for the histograms for this dataset). From Figure 4, we can see the upper tails of the histograms of the two groups are different. Similar results can be found in Figure 5.

Results (p values of tests) are shown in Table 6. As can be seen, when setting *τ *equal to 110 (100) for the first (second) datasets, both the methods for tests of *H*_0,*A *_and the new methods for tests of *H*_0,*C *_can detect the differences in 'maximum lifespan' between groups at nominal alpha levels of 0.01 (0.05) for the first (second) datasets. But the methods for tests of *H*_0,*B *_cannot detect the difference for all different values of *τ *. The following description may provide some explanation to these results. For the first dataset, when set *τ *= 110, the proportions of the observations greater than *τ *in the EO-HF group and WL-HF group (i.e., estimations of *P*(*Y *> *τ *| *X *= 0) and *P*(*Y *> *τ *| *X *= 1)) are 0.061 and 0.306, respectively. These two proportions are significantly different and not surprisingly, the methods for tests of *H*_0,*A *_can detect the difference in 'maximum lifespan' between the two groups. Second, the sample means of the observations greater than *τ *in the two groups (i.e., estimations of *μ *(*Y *| *Y *> *τ *∩ *X *= 1) and *μ *(*Y *| *Y *> *τ *∩ *X *= 0)) are 117.8 and 122.9, respectively, and there is no much difference between these sample means. However the sample means of the Z-values in the two group (i.e., the estimations of *P*(*Z *| *X *= 0) and *P*(*Z *| *X *= 1)) are 7.210 and 37.633, respectively, and are *greatly *different, where, *Z*_*i *_≡ *I*(*Y*_*i *_> *τ*)*Y*_*i*_. These may explain that the methods for tests of *H*_0,*B *_cannot reject the null but the new methods for tests of *H*_0,*C *_can detect the difference in 'maximum lifespan' between the two groups. Similarly, for the second dataset, when set *τ *= 100, the proportions of the observations greater than *τ *in the group with genotype '+/+' and group with genotype '-/-' are 0.188 and 0.571, respectively. The sample means of the observations greater than *τ *in the two groups are 109.3 and 110.9, respectively. The sample means of the Z-values in the two groups are 20.5 and 63.4 respectively.

**Table 6:** Results (p values of tests) of application to two real datasets.

**Test**	**Data from Vasselli et al. **[10]^**1**^	**Data from Redmann & Argyropoulos **[14]^**2**^
***Tests of H*_**0,*A ***_*(Wang-Allison tests)***		
QT3 with *τ *set to 110/100^#^.	0.002	**0.027**
QT3 with *τ *set to sample 90^th ^percentile.	0.038	**0.186**
QT4 with *τ *set to 110/100.	0.002	**0.022**
QT4 with *τ *set to sample 90^th ^percentile.	0.033	**0.146**
***Tests of H_**0,*B***_***		
Wilcoxon-Mann-Whitney with *τ *set to 110/100.	0.289	**0.868**
Wilcoxon-Mann-Whitney with *τ *set to sample 90^th ^percentile.	0.750	N/A*
Permutation test with *τ *set to 110/100.	0.281	**0.738**
Permutation test with *τ *set to sample 90^th ^percentile.	0.634	N/A*
***Tests of H*_**0,*C ***_*(new tests)***		
Wilcoxon-Mann-Whitney with *τ *set to 110/100.	0.001	**0.022**
Wilcoxon-Mann-Whitney with *τ *set to sample 90^th ^percentile.	0.026	**0.243**
Permutation test with *τ *set to 110/100.	0.001	**0.014**
Permutation test with *τ *set to sample 90^th ^percentile.	**0.024**	**0.072**

Notes: In each dataset, males and females have been combined. ^1^For the data from Vasselli et al. [10] two groups of rats (EO-HF and WL-HF) are compared; each group has 49 observations.

^2^The data from Redmann & Argyropoulos [14] consists of 16 mice with genotype '+/+' and 21 mice with genotype '-/-'.

^# ^For Data from Vasselli et al. [10]*τ *is set to 110; for data from Redmann & Argyropoulos [14]*τ *is set to 100.

*Only one group has observations above the threshold *τ*.

From Table 6 we can also see that in almost all situations the p-values of the new methods for tests of *H*_0,*C *_are somewhat smaller than those of the methods for tests of *H*_0,*A*_. This is consistent with the simulations showing greater power of the new methods.

## Discussion

Herein, we proposed new methods for testing the difference of 'maximum' lifespan between groups (e.g., treatment and control). By defining a new variable *Z *such that *Z*_*i *_≡ *I *(*Y*_*i *_> *τ*)*Y*_*i *_for each observation and then applying Wilcoxon-Mann-Whitney test or better still a permutation test to *Z*, the new methods achieve far better performance when considered across a broad range of circumstances in terms of both Type-1 error rates and power. In the new methods, we use the Wilcoxon-Mann-Whitney test or permutation test. One could also choose to use a bootstrap test in place of these two tests. However, additional simulations would likely be warranted to evaluate its performance relative to the permutation test we have evaluated herein.

It is straightforward to extend the new methods to more than two groups. For example, one could use the Kruskal-Wallis Test to replace the Wilcoxon-Mann-Whitney test, or use permutation testing for multiple groups to replace that for two groups.

We have shown that the new methods are effective by simulation studies when the sample size (N) of each group is 50, 100, or 200. We expect that these methods will be also be relatively more powerful than existing competitors for much larger sample sizes, such as N = 500 or even N = 5000. There are some mouse data sets (like those of the National Institute of Aging's Intervention Testing Program) where N > 500, and worm and fly data sets in which N may sometimes even exceed 5000. We expect that the new methods are equally applicable to the analysis of such data.

Finally, we note that the tests proposed here are described for the context of testing for differences in lifespan. However, there is nothing intrinsic to them that limits their use to survival data. They could be applied to any situation in which one wanted to test for group differences in the tails of distributions.

## Competing interests

The authors declare they have no competing interests.

## Authors' contributions

DBA participated in all parts of the work of the study (including the study design, methodology development, simulations, data acquisition, and manuscript drafting). He wrote major sections of the original manuscript. He revised final version of the manuscript. DTR provided consulting on the statistical issues in the study and manuscript editing. SZ provided assistance in programming for simulation studies. WW provided consulting on simulation and prepared the figures. GG did all simulation studies and real data analyses and drafted the sections of Results, Illustration with real data, and Discussion of the manuscript and participated in revision of the manuscript.

## Pre-publication history

The pre-publication history for this paper can be accessed here:


